# JME-001 phase II trial of first-line combination chemotherapy with cisplatin, pemetrexed, and nivolumab for unresectable malignant pleural mesothelioma

**DOI:** 10.1136/jitc-2021-003288

**Published:** 2021-10-28

**Authors:** Yosuke Miyamoto, Toshiyuki Kozuki, Keisuke Aoe, Sae Wada, Daijiro Harada, Michihiro Yoshida, Jun Sakurai, Katsuyuki Hotta, Nobukazu Fujimoto

**Affiliations:** 1 Department of Medical Oncology, Okayama Rosai Hospital, Okayama, Japan; 2 Department of Thoracic Oncology and Medicine, National Hospital Organization Shikoku Cancer Center, Matsuyama, Japan; 3 Department of Medical Oncology, National Hospital Organization Yamaguchi-Ube Medical Center, Ube, Japan; 4 Center of Innovative Clinical Medicine, Okayama University Hospital, Okayama, Japan

**Keywords:** clinical trials, phase II as topic

## Abstract

**Background:**

JME-001 is a phase II trial assessing the efficacy and safety of cisplatin, pemetrexed, and nivolumab as first-line therapy in malignant pleural mesothelioma (MPM).

**Patients and methods:**

Patients with untreated, unresectable MPM with an Eastern Cooperative Oncology Group (ECOG) performance status (PS) of 0–1 were included. The primary endpoint is the centrally reviewed objective response rate. The secondary endpoints include (1) response rate assessed by investigators, (2) disease control rate, (3) overall survival, (4) progression-free survival, (5) duration of response, and (6) time to response. Safety and adverse events will also be evaluated. Cisplatin (75 mg/m^2^), pemetrexed (500 mg/m^2^), and nivolumab (360 mg/body) were administered intravenously every 3 weeks with a total of 4–6 cycles. If patients did not progress during the combination phase, maintenance therapy with nivolumab was administered until disease progression or unacceptable toxicity. Tissue samples were required and collected for programmed death ligand 1 analysis.

**Results:**

Eighteen patients (mean age 69.2 years, 15 men) were enrolled between January 2018 and May 2019. The ECOG PS was 0 in 3 patients and 1 in 15 patients. Fourteen (77.8%; 95% CI 52.4% to 93.6%) patients had an objective response. The disease control rate was 94.4% (95% CI 72.7% to 99.9%). Fourteen (77.8%) patients had partial response (PR), three had stable disease, and one was not evaluable. Tumor shrinkage was observed in 10/14 (71.4%) patients with epithelioid, and 2/2 (100%) patients with sarcomatoid or biphasic histological subtype had PR. Ten (55.6%) patients experienced grade 3 or worse adverse events, including disorder of metabolism or nutrition (33.3%), loss of appetite (27.8%), anemia (16.7%), and hyponatremia (11.1%). No treatment-related deaths occurred.

**Conclusions:**

The safety and efficacy of this study strongly support a definitive trial of this combination.

**Trial registration number**

UMIN000030892.

## Introduction

Malignant pleural mesothelioma (MPM) is an aggressive tumor that arises from mesothelial-lined surfaces and has a poor survival rate.^1^ The industrial use of asbestos has been banned in Japan since 2006, but the incidence of MPM is expected to continue to increase for the next few decades due to past usage of asbestos.^2^ Treatment of MPM is challenging. Most cases are diagnosed at an advanced stage and treated with systemic chemotherapy. Combination chemotherapy with cisplatin and pemetrexed is the standard treatment regimen; however, the median overall survival (OS) is only about 12 months.^3^ Recently, the addition of bevacizumab was shown to improve OS when added to cisplatin and pemetrexed in the treatment of unresectable MPM.^4^ However, the prolongation of OS was less than 3 months and it can only be administered to bevacizumab-eligible patients. Therefore, cisplatin and pemetrexed is still considered the standard treatment regimen and additional treatment options are urgently needed.

Immune checkpoint inhibitors (ICIs), such as programmed death-1 (PD-1), programmed death-ligand 1 (PD-L1), and cytotoxic T lymphocyte associated protein-4 (CTLA-4), have revolutionized cancer treatment. Nivolumab is a human monoclonal antibody that targets the PD-1 cluster of differentiation 279 cell surface membrane receptor. Binding of PD-1 to its ligands, PD-L1 and PD-L2, results in the downregulation of lymphocyte activation. Nivolumab inhibits the interaction between PD-1 and its ligands, promotes immune responses, and triggers antitumor activity and has already been approved in Japan for multiple types of cancer, including malignant melanoma, non-small cell lung cancer, and gastric cancer. Mesothelioma carcinogenesis occurs on the background of the chronic inflammatory responses to asbestos, and the tumor microenvironment is composed of pro-inflammatory cytokines, growth factors, endothelial cells, stromal cells, and immune cells.^5^ Thus, there is a strong biological rationale to use ICIs in MPM. A phase II trial has demonstrated a favorable response to nivolumab in previously treated MPM.^6^ Based on the results, nivolumab has been approved for patients with MPM that is refractory or intolerable to platinum/pemetrexed chemotherapy.

A recent report indicated that platinum drugs enhance the effector immune response through modulation of PD-L1.^7^ These encouraging results may extend to the first-line treatment of MPM with the hope of enhancing the antitumor response, particularly when used in combination with the current standard chemotherapy. Unfortunately, no prospective clinical trial is being conducted to evaluate the combination of nivolumab and cisplatin/pemetrexed. Therefore, we launched the current trial to assess combination chemotherapy with cisplatin, pemetrexed, and nivolumab for MPM.

## Materials and methods

### Study design and patients

JME-001 is a single-arm, prospective, non-randomized, non-comparative, open label, multicenter, phase II trial conducted from January 1, 2018, to November 30, 2019 (data cut-off date), at four centers in Japan. All patients who met the inclusion and exclusion criteria (online supplemental tables 1 and 2) were invited for screening. Eligible patients were ≥20 years old with histologically confirmed, untreated, unresectable advanced MPM and had ≥1 measurable lesion(s) as defined in the modified Response Evaluation Criteria in Solid Tumors V.1.1 (mRECIST)^8^ for mesothelioma and confirmed by imaging within 14 days prior to enrollment. Eligible patients also had to have tumor tissue samples available for the analysis of PD-L1 expression and an Eastern Cooperative Oncology Group (ECOG) performance status of 0 or 1. Main exclusion criteria were severe hypersensitivity reactions to any other drug, including antibody products; concurrent autoimmune disease or a history of chronic or recurrent autoimmune disease; multiple primary cancers; brain metastases; current or history of interstitial lung disease or pulmonary fibrosis diagnosed based on imaging or clinical findings; or previous treatment with nivolumab, anti-PD-1 antibody, anti-PD-L1 or PD-L2, or any other therapeutic antibodies or pharmacotherapies for T-cell regulation.

10.1136/jitc-2021-003288.supp3 Supplementary data

### Procedures

Treatment comprised two sequential phases: the combination phase and the maintenance phase. In the combination phase, cisplatin (75 mg/m^2^), pemetrexed (500 mg/m^2^), and nivolumab (360 mg/body) were administered intravenously. Nivolumab was kindly provided by Ono Pharmaceutical. This treatment was mandated to repeat every 3 weeks for a total of 4–6 cycles. If there was no progression of MPM during the combination phase, maintenance therapy with nivolumab was administered until disease progression, unacceptable toxicity, or the patient’s condition met the withdrawal criteria.

Both cisplatin and pemetrexed are usually administered every 3 weeks. Under the consideration of practical utility and dose intensity, we planned to administer nivolumab every 3 weeks at the dose of 360 mg/body. Patients underwent tumor imaging by CT or MRI every three cycles. Target lesion diameters were measured, and the tumor response was assessed according to mRECIST criteria.

PD-L1 expression was analyzed in a central laboratory (Cancer Genetics, New Jersey, USA) using archival tumor tissue samples with 28–8 antibody (Dako, California). One or more formalin-fixed, paraffin-embedded (FFPE) blocks of tumor tissue samples collected by core needle biopsy, excisional biopsy, or incisional biopsy of ≥5 FFPE unstained slide samples (serial tissue sections) were analyzed for PD-L1 status. Each sample was required to contain ≥100 evaluable tumor cells. PD-L1-positive was defined as membranous staining in ≥1% of tumor cells. Samples were classified as not evaluable (NE) if the biological conditions of the sample rendered the stained cell membranes difficult to assess, even if the samples otherwise met the evaluation criteria.

### Outcomes

This study assessed the efficacy and safety of first-line combination therapy with cisplatin, pemetrexed, and nivolumab for advanced or metastatic MPM. The primary endpoint was the centrally assessed objective response according to mRECIST. The objective response rate (ORR) was defined as the proportion of patients whose best overall response was a complete response (CR) or partial response (PR). The secondary endpoints included efficacy evaluated by the (1) response rate assessed by investigators, (2) disease control rate, (3) OS, (4) progression-free survival (PFS), (5) response duration, and (6) time to response. Safety and adverse events were also evaluated.

The OS was defined as the duration from study registration until the date of death from any cause. PFS was defined as the time from registration to first progressive disease (PD) or death from any cause, whichever is earlier. The disease control rate was the percentage of patients whose best overall response was CR, PR, or stable disease (SD).

Adverse events (AEs) and treatment-related AEs (TRAEs) were monitored throughout the study period and graded according to the National Cancer Institute Common Terminology Criteria for Adverse Events, V.4.0. Quality of life (QOL) was evaluated using the EuroQol 5 Dimension Japanese edition^9^ and the Lung Cancer Symptom Scale for Mesothelioma.^10^ QOL was evaluated at each treatment visit according to the treatment schedule before the administration of agents.

### Statistical analysis

The trial size was set as 18 due to feasibility. If we assume that 6–12 patients would have a response, the response rate would be 33.3%–66.7%. In this case, the estimate accuracy indicates that the range between the point estimate of the response rate and the lower confidence limit (two-sided 95% confidence coefficient based on exact test) would be 18%–22%.

The statistical analysis was conducted based on predetermined statistical analysis plan. The efficacy and safety-related endpoints were analyzed with full and safety analysis sets, respectively. The patient characteristics, the numbers of treatment cycles and dose reductions, duration of treatment, the relative dose intensity and trial continue/discontinue condition with the reasons were summarized. The centrally reviewed ORR (primary endpoint), investigator-assessed ORR and the disease control rate (included in the secondary endpoints) were estimated with 95% CI. Response rate per histological subtypes and PD-L1 expression status were also calculated. The other secondary endpoints; OS, PFS, duration of response, and time to response were analyzed based on the Kaplan-Meier product limit approach. The best reduction percentage and the change in the sum of target lesions from baseline in each patient were graphed (waterfall and spider plots). The frequency of AEs and TRAEs were summarized with the grade. The summary statistics of the QOL scale/score difference between time points was calculated.

### Role of the funding source

The funding source was not involved in the study design, the collection, analysis, and interpretation of data, writing the report, or in the decision to submit the paper for publication.

## Results

Eighteen patients were enrolled between January 2018 and May 2019 (table 1). Four patients (22.2%) continued treatment, and 14 (77.8%) discontinued treatment until data cut-off (November 30, 2019). The reasons for discontinuation included PD (n=8, 44.4%), development of a grade 3 or greater infusion reaction (n=1, 5.6%), and continuation of treatment judged as inappropriate by the principal investigator (n=3, 16.7%). One patient withdrew consent to the treatment after the first cycle of induction chemotherapy. All 18 patients were included in both the full and safety analysis sets. Median follow-up was 15.2 (range 6.9–19.4) months.

**Table 1:** Patient characteristics (N=18)

Characteristic	Value
Median age, years (range)	69 (64–78)
Male/female	15 (83) / 3 (17)
ECOG PS, 0/1	3 (17) / 15 (83)
Histological subtype	
Epithelioid	14 (77.8)
Sarcomatoid	2 (11.1)
Biphasic	2 (11.1)
TNM classification	
T1N0M0	2 (11.1)
T1N2M0	1 (5.6)
T2N0M0	1 (5.6)
T3N0M0	6 (33.3)
T3N2M0	1 (5.6)
T4N0M0	3 (16.7)
T4N1M0	1 (5.6)
T4N2M0	2 (11.1)
T4N2M1	1 (5.6)
Stage	
I	8 (44.4)
II	0 (0.0)
III	9 (50)
IV	1 (5.6)
PD-L1 expression	
<1%	1 (5.6)
≥1%	17 (94.4)

Values are n (%) unless otherwise noted.

ECOG, Eastern Cooperative Oncology Group; PD-L1, programmed death-ligand 1; PS, performance status; TNM, tumor, node, metastases.

Patients received an average of 4.8 (range 2–6) cycles of induction triplet chemotherapy. Nine patients (50.0%) received four cycles and eight patients (44.4%) received six cycles. The average number of dose reductions was 0 for nivolumab, 0.3 (range 0–1) for pemetrexed, and 0.3 (range 0–1) for cisplatin. The relative dose intensity in combination phase was 93.5% (range 75.0%–100.0%) for nivolumab, 89.4% (range 60.9%–101.3%) for pemetrexed, and 90.1% (range 63.8%–101.1%) for cisplatin. The average number of nivolumab total cycles was 10.9 (range 2–26). The average total duration of treatment was 7.5 (range 0.7–18.7) months.

**Figure 1:**
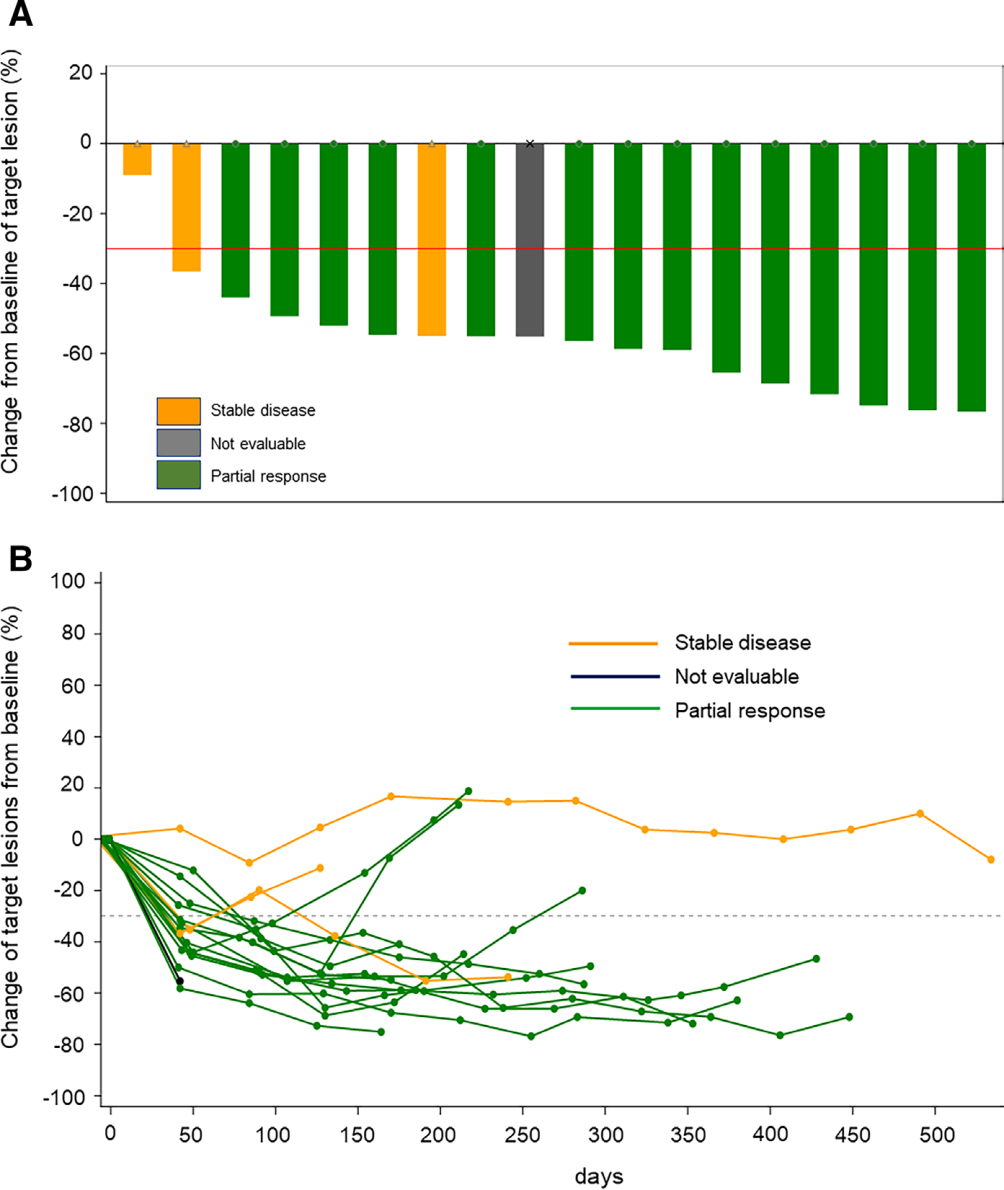
The best reduction percentage (A) and the change in the of sum of target lesions (B) in each patient.

The best percentage reduction and the change in the sum of target lesions in each patient are shown in figure 1. Fourteen (77.8%; 95% CI, 52.4% to 93.6%) patients had an objective response by central assessment (table 2), which was consistent with the investigator-assessed objective response. Regarding best overall response, 14 patients had a PR. The responses and disease control rate are given in table 2. Tumor shrinkage was observed in all histological subtypes, in 10/14 (71.4%) patients with epithelioid, and the four patients with non-epithelioid disease had a PR. The three remaining patients with epithelioid had SD and one remaining patient with epithelioid was NE. Tumor shrinkage was observed regardless of PD-L1 status and occurred in 13/17 (76.5%) patients with PD-L1 expression ≥1% and 1/1 (100%) patients with PD-L1 expression <1%.

**Table 2:** Response and disease control rates

	No. of patients	%
Response		
CR	0	0
PR	14	77.8
SD	3	16.7
PD	0	0
NE	1	5.6
Response rate (95% CI)		77.8 (52.4 to 93.6)
Disease control rate (95% CI)		94.4 (72.7 to 99.9)

CR, complete response; NE, not evaluable; PD, progressive disease; PR, partial response; SD, stable disease.

At data cut-off, three patients (16.7%) had an ongoing response. The median response duration was 6.7 months (95% CI 4.21 to not reached), with median time to response of 1.54 (range 1.4–3.3) months. The median reduction in target lesions from baseline (depth of response) was 55.9% (IQR 52.2%–68.8%).

The Kaplan-Meier curve for PFS, determined by blinded independent central review, is shown in online supplemental figure 1A. At the time of data cut-off, 11 (61.1%) events had occurred, and 7 (38.9%) cases were censored. All 11 events were PD, and there was no death before disease progression. Median PFS was 8.02 months (95% CI 5.59 to 14.06). The 6-month and 12-month PFS rate was 69.0% (95% CI 40.8% to 85.8%) and 40.3% (95% CI 16.2% to 63.5%), respectively. The Kaplan-Meier curve for OS is shown in online supplemental figure 1B. At the time of data cut-off, 2 (11.1%) events had occurred, and 16 (88.9%) cases were censored. Median OS was 20.8 months. The 6-month and 12-month overall survival rate was 100% (95% CI 100.0% to 100.0%) and 92.3% (95% CI 56.6% to 98.9%), respectively.

10.1136/jitc-2021-003288.supp1 Supplementary data

10.1136/jitc-2021-003288.supp2 Supplementary data

All 18 patients experienced AEs, but no treatment-related death was recorded. All-cause AEs occurring in ≥10% of patients are shown in table 3. Ten (55.6%) patients experienced grade 3 or greater AEs, including disorder of metabolism or nutrition, loss of appetite, anemia, hyponatremia, leukopenia, lymphocytopenia, increased serum alanine aminotransferase, increased serum aspartate aminotransferase, pneumonia, nausea, colitis, diverticulitis, dental pulpitis, pulmonary embolism, peripheral neuropathy, and back pain. Two patients (11.1%) experienced peripheral neuropathy during nivolumab maintenance treatment, leading to treatment discontinuation.

**Table 3:** Adverse events

Event	Grade 1	Grade 2	Grade 3	Grade 4	Unknown	Total	Grade ≥3
Nausea	7 (38.9)	4 (22.2)	1 (5.6)	–	–	12 (66.7)	1 (5.6)
Appetite loss	2 (11.1)	4 (22.2)	5 (27.8)	–	–	11 (61.1)	5 (27.8)
Hiccup	4 (22.2)	7 (38.9)	–	–	–	11 (61.1)	–
Constipation	4 (22.2)	5 (27.8)	–	–	–	9 (50.0)	–
Rush	3 (16.7)	4 (22.2)	–	–	–	7 (38.9)	–
Anemia	–	4 (22.2)	3 (16.7)	–	–	7 (38.9)	3 (16.7)
Fatigue	2 (11.1)	4 (22.2)	–	–	–	6 (33.3)	–
Nasopharyngitis	3 (16.7)	2 (11.1)	–	–	–	5 (27.8)	–
Insomnia	4 (22.2)	1 (5.6)				5 (27.8)	–
Neutropenia	–	5 (27.8)	–	–	–	5 (27.8)	–
Diarrhea	1 (5.6)	3 (16.7)	–	–	–	4 (22.2)	–
Fever	4 (22.2)	–	–	–	–	4 (22.2)	–
Peripheral neuropathy	2 (11.1)	1 (5.6)	1 (5.6)		–	4 (22.2)	1 (5.6)
Leukopenia	–	3 (16.7)	1 (5.6)	–	–	4 (22.2)	1 (5.6)
Mucositis	–	3 (16.7)	–	–	–	3 (16.7)	–
Pneumonia	–	2 (11.1)	1 (5.6)	–	–	3 (16.7)	1 (5.6)
Dysgeusia	1 (5.6)	1 (5.6)	–	–	1 (5.6)	3 (16.7)	–
Hearing impairment	2 (11.1)	1 (5.6)	–	–	–	3 (16.7)	–
Abdominal discomfort	1 (5.6)	1 (5.6)	–	–	–	2 (11.1)	–
Angular cheilitis	1 (5.6)	1 (5.6)	–	–	–	2 (11.1)	–
Hyponatremia	–	–	1 (5.6)	1 (5.6)	–	2 (11.1)	2 (11.1)
Muscle pain	1 (5.6)	1 (5.6)	–	–	–	2 (11.1)	–
Back pain	–	1 (5.6)	1 (5.6)	–	–	2 (11.1)	1 (5.6)

Values are n (%).

The mean (±SD) difference in the health visual analog scale based on the start of the induction treatment was −5.6±24.2 mm (range −65 to 30) at the start of nivolumab maintenance treatment and 0.5±23.3 mm (range −40 to 30) at the end of the treatment. The mean (±SD) difference in the index score based on the start of the induction treatment was 0.0185±0.1389 (range −0.319 to 0.292) at the start of nivolumab maintenance treatment and −0.0166±0.1912 (−0.364 to 0.292) at the end of the treatment. The mean (±SD) difference in the total visual analog scale based on the start of the induction treatment was −0.01±13.57 mm (−21.1 to 28.1) at the start of nivolumab maintenance treatment and −2.11±21.38 mm (−41.3 to 36.0) at the end of the treatment.

## Discussion

To the best of our knowledge, this study is the first clinical trial to evaluate the effect of combining nivolumab and platinum-based chemotherapy for the treatment of advanced MPM. The combination of an ICI and cytotoxic chemotherapy is a rapidly evolving area of interest in cancer treatment. Cytotoxic agents, including platinum, could modulate the immune response through PD-1/PD-L1 inhibition by enhancing the potential immunogenic effect.^11–13^ Combination regimens that include a PD-1 or PD-L1 inhibitor have led to prolonged OS in small cell lung cancer^14^ and non-small cell lung cancer.^15^ Previous reports have also shown that cytotoxic agents can induce immune-stimulating properties in mesothelioma cell models.^16 17^

Nivolumab is currently administered at a dose of 240 mg/body biweekly in clinical practice based on recent clinical trials.^6 18^ However, combination chemotherapy with cisplatin and pemetrexed is administered every 3 weeks. In the current study, nivolumab was administered every 3 weeks at a dose of 360 mg/body based on a recent report that the combination of nivolumab (10 mg/kg) and pemetrexed/cisplatin every 3 weeks has an acceptable toxicity profile and encouraging antitumor activity in patients with advanced non-small cell lung cancer.^19^

We set a centrally assessed ORR according to mRECIST as the primary endpoint. A modification of the RECIST criteria has specifically addressed the difficulties measuring and assessing changes in tumor bulk in MPM. In addition, the mRECIST criteria have successfully distinguished between responders and non-responders for the parameters of OS,^20^ demonstrating its ability as an appropriate endpoint, particularly in phase II studies. The combination of nivolumab and cisplatin/pemetrexed has demonstrated a notable ORR of 77.8%. This is the highest ORR reported thus far in chemotherapy for MPM. Moreover, all participants demonstrated tumor shrinkage. One of the most remarkable aspects of the participants in the current study was a high tumor proportion score for PD-L1 expression. PD-L1 is expressed in a substantial proportion of MPM and is associated with poor survival.^21^ The association of PD-L1 expression in mesothelioma cells and the response to anti-PD-1 inhibitors are still controversial. PD-L1 positivity was not correlated with outcome in one trial,^22^ but increased ORR and prolonged survival was observed in patients with PD-L1-positive patients in another study.^6^ Nivolumab plus ipilimumab combination therapy exhibited higher ORR in patients with PD-L1-positive MPM compared with that in patients with PD-L1-negative MPM.^23^ In another study, PD-L1 expression was not only associated with the increase of ORR but also associated with the improvement in PFS and OS when treated with a combination of nivolumab plus ipilimumab.^24^ These results indicate that PD-L1 expression could be a reliable biomarker for ICI response. The high PD-L1 expression may contribute to the favorable response in the current study. The AE profile in the current study was consistent with what is expected when combining cisplatin and pemetrexed with nivolumab. The addition of nivolumab did not appear to increase the frequency or severity of AEs associated with chemotherapy with cisplatin and pemetrexed.

Recently, a multicenter phase II study was conducted in Australia^25^ in 55 patients with untreated MPM who received cisplatin, pemetrexed, and durvalumab for a maximum of six cycles, followed by durvalumab maintenance for up to 12 months. The primary endpoint, 6-month PFS, was 57%, and the ORR and disease control rate were 48% and 87%, respectively. Based on these favorable results, a multicenter trial is planned to randomize participants for cisplatin and pemetrexed with or without durvalumab. More recently, an international randomized phase III trial evaluated the combination of ipilimumab, a CTLA-4 inhibitor, and nivolumab versus standard first-line platinum-pemetrexed chemotherapy in treatment-naïve patients with untreated, unresectable MPM.^26^ The primary endpoint of OS was met with a 4-month prolongation in median OS in those who received nivolumab–ipilimumab compared with those who received platinum–pemetrexed chemotherapy. These findings led to the recent approval of nivolumab plus ipilimumab in the USA for first-line treatment of unresectable MPM. The combination of nivolumab and ipilimumab would be a new standard first-line treatment, but some problems still remain. One of the problems is a rapid drop-off in PFS in patients receiving nivolumab plus ipilimumab. Similar results have been shown in clinical trials of non-small cell lung cancer, which has shown improvement in OS and PFS.^27^ A recent study of non-small cell lung cancer that ipilimumab plus nivolumab with two cycles cytotoxic chemotherapy demonstrated an improvement in the rapid drop-off of PFS and OS.^28^ These results support the further clinical development of the ICI-chemotherapy combination in first-line treatment of MPM.

The main limitation of the current study is its single-arm, non-comparative design. In addition, we included a few participants without tumor PD-L1 expression. Survival analyses are immature because most of the participants were censored at the time of data cut-off. The trial size was determined based not on statistical power, but on our ability to accrue patient. However, the estimated lower limit of the ORR in the current study was 52.4%, which is higher than the ORRs reported in previous studies of front-line cisplatin/pemetrexed combination chemotherapy.

In conclusion, the combination of cisplatin, pemetrexed, and nivolumab demonstrated sufficient activity and safety as first-line therapy in unresectable MPM. We think that adding nivolumab to cisplatin/pemetrexed would be a treatment option for patients with advanced MPM, though the efficacy and safety should be examined in a definitive randomized study.

## Data Availability

Data are available upon reasonable request. The patients’ de-identified clinical data may be made available to other investigators after approval by the institutional review board. Requests should be directed to the corresponding author.
