# Supplementary material for: JME-001 phase II trial of first-line combination chemotherapy with cisplatin, pemetrexed, and nivolumab for unresectable malignant pleural mesothelioma
Source: J Immunother Cancer. 2021 Oct 28;9(10):e003288. doi: 10.1136/jitc-2021-003288 (PMC8557301; doi:10.1136/jitc-2021-003288)
Supplement: Supplementary data [file jitc-2021-003288supp003.pdf]

Supplementary Table 1. Key inclusion criteria

1) Age >20 years at the date of informed consent
2) Pathologically confirmed pleural malignant mesothelioma
3) Advanced or metastatic malignant pleural mesothelioma that is untreated and unresectable
4) A measurable lesion designated by modified RECIST criteria
5) Tumor sample available to test for programmed death-ligand 1 expression
6) Eastern Cooperative Oncology Group Performance Status 0 or 1
7) Life expectancy is ≥ 90 days
8) Oxygen saturation measured by pulse oximeter is ≥ 94%
9) Meet the defined lab criteria
10) Females of child-bearing potential who agree to prevent pregnancy and lactation for at least 5 months after the last administration of nivolumab
11) Males who agree to contraception for at least 7 months after the last administration of nivolumab
12) Patients who understand the study information and provide written consent by their own free will

RECIST=Response Evaluation Criteria in Solid Tumors

Supplementary Table 2. Key exclusion criteria

1) History of anaphylaxis induced by any drug
2) Autoimmune disease
3) Double cancer
4) Metastasis to the brain or meninges
5) Interstitial lung disease or pulmonary fibrosis
6) Diverticulitis or peptic ulcer
7) Pleural effusion that requires drainage every 2 weeks or more
8) Pericardial effusion or ascites that requires drainage
9) Uncontrollable cancer pain
10) Transient ischemic attack, cerebrovascular accident, thrombosis, or thromboembolism within 180 days
11) Uncontrollable severe cardiovascular disease
12) Anticoagulant therapy
13) Uncontrollable diabetes
14) Receiving treatment for a systemic infection
15) Obviously positive for Human Immunodeficiency Virus
16) HTLV-1 antibody positive, HBs antigen positive, or HCV antibody positive. Either HBs antigen positive or HBc antibody positive and HBV-DNA detection if HBs antigen is negative.
17) History of treatment for T-cell regulation
18) Surgery with local or surface anesthesia within 14 days
19) Surgery with general anesthesia within 28 days
20) Pleurodesis within 14 days
21) Pleurodesis treated with Picibanil within 28 days
22) Adhesion surgery of the pericardium or peritoneum
23) Radiation therapy for pain relief within 14 days
24) Radiopharmaceutical therapy within 56 days
25) Administration of unapproved drugs within 28 days or an unapproved antibody within 90 days
26) Administration of systemic adrenal cortical hormone or immunosuppressive agents
27) Females who are or may be pregnant or lactating
28) Patients who are incapable of giving consent (e.g., due to dementia)
29) Any other inadequacy for this study

HTLV=human T-cell leukemia virus, HB=hepatitis B, HCV=hepatitis C virus
