# Supplementary figures and images for: JME-001 phase II trial of first-line combination chemotherapy with cisplatin, pemetrexed, and nivolumab for unresectable malignant pleural mesothelioma
Source: J Immunother Cancer. 2021 Oct 28;9(10):e003288. doi: 10.1136/jitc-2021-003288 (PMC8557301; doi:10.1136/jitc-2021-003288)

Supplementary Figure 1 (A)

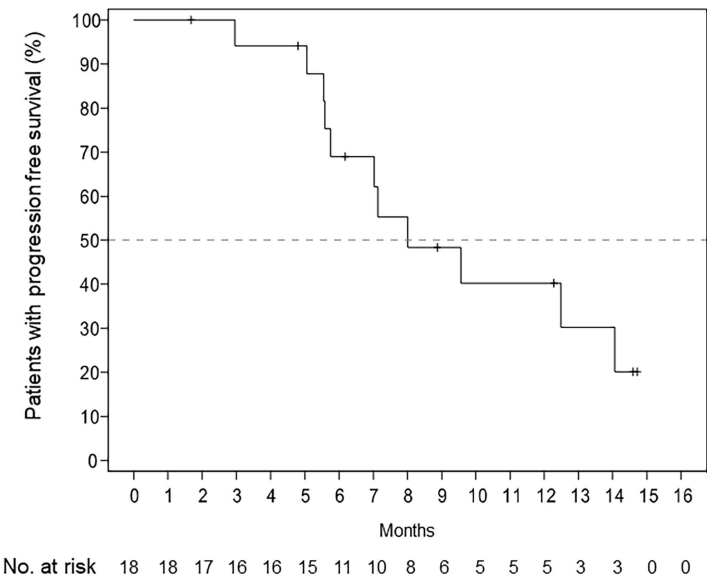

Supplement: Supplementary data [file jitc-2021-003288supp001.pdf]

Supplementary Figure 1 (B)

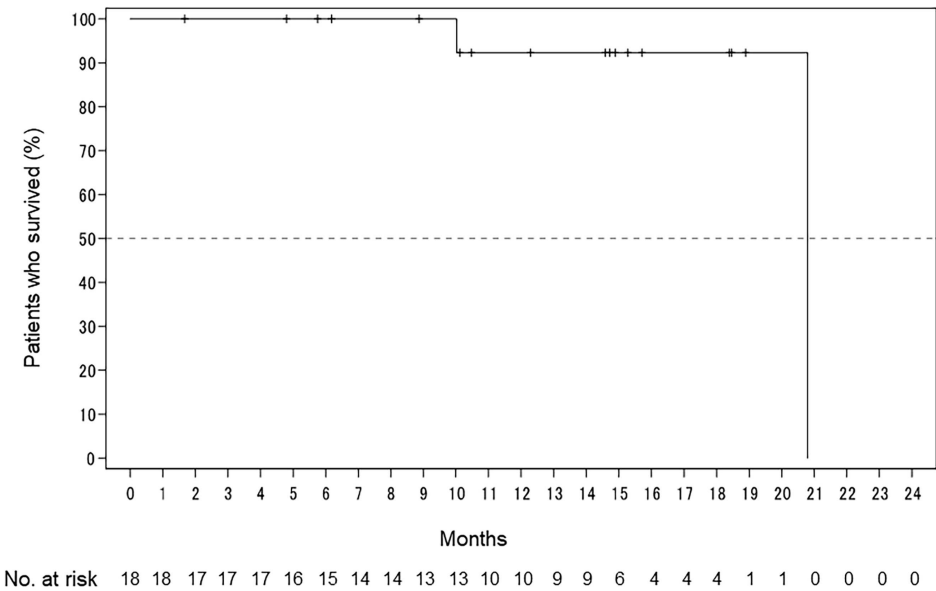

Supplement: Supplementary data [file jitc-2021-003288supp002.pdf]
